# Intracortical Circuits in the Contralesional Primary Motor Cortex in Patients With Chronic Stroke After Botulinum Toxin Type A Injection: Case Studies

**DOI:** 10.3389/fnhum.2020.00342

**Published:** 2020-08-24

**Authors:** Maryam Zoghi, Pouya Hafezi, Bhasker Amatya, Fary Khan, Mary Pauline Galea

**Affiliations:** ^1^Department of Physiotherapy, Podiatry, Prosthetics and Orthotics, La Trobe University, Melbourne, VIC, Australia; ^2^Monash Health, Melbourne, VIC, Australia; ^3^The Royal Melbourne Hospital, Melbourne, VIC, Australia; ^4^University of Melbourne, Melbourne, VIC, Australia

**Keywords:** stroke, primary motor cortex, contralesional, case studies, intracortical circuits, spastic, botulinum A toxin

## Abstract

Spasticity and motor recovery are both related to neural plasticity after stroke. A balance of activity in the primary motor cortex (M1) in both hemispheres is essential for functional recovery. In this study, we assessed the intracortical inhibitory and facilitatory circuits in the contralesional M1 area in four patients with severe upper limb spasticity after chronic stroke and treated with botulinum toxin-A (BoNT-A) injection and 12 weeks of upper limb rehabilitation. There was little to no change in the level of spasticity post-injection, and only one participant experienced a small improvement in arm function. All reported improvements in quality of life. However, the levels of intracortical inhibition and facilitation in the contralesional hemisphere were different at baseline for all four participants, and there was no clear pattern in the response to the intervention. Further investigation is needed to understand how BoNT-A injections affect inhibitory and facilitatory circuits in the contralesional hemisphere, the severity of spasticity, and functional improvement.

## Introduction

Spasticity and weakness (spastic paresis) are the primary motor impairments after stroke and impose significant challenges for treatment and patient care. Spasticity not only has downstream effects on the patient’s quality of life but also places substantial burdens on the caregivers and society (Zorowitz et al., 2013). Spasticity and motor recovery are both related to neural plasticity after stroke. There is considerable variability in the onset of spasticity, which may occur in the short-, medium- or long-term after stroke (Ward, 2012), and the relationship between spasticity and motor recovery remains poorly understood by clinicians and researchers. There is evidence to suggest that spasticity, as a post-stroke deficit, is associated with reorganization of structures in the central nervous system in a way that is detrimental to stroke recovery (so-called maladaptive plasticity) (Pascual-Leone et al., 2005). Facilitation and modulation of neural plasticity through rehabilitative strategies, such as early intervention with repetitive goal-oriented intensive therapy, appropriate non-invasive brain stimulation, and pharmacological agents, are the keys to promoting motor recovery.

Cortical reorganization may occur in both hemispheres during the acute stages after stroke, representing an adaptive process that enables latent motor pathways to become active. Functional MRI studies have shown that there is widespread brain activation early after stroke, which reduces over subsequent months (Ward et al., 2003; Grefkes and Fink, 2011). A balance of activity in the primary motor cortex (M1) in both hemispheres is essential for functional recovery. In the chronic stages after stroke, there is an imbalance of hemispheric activity, with several studies using transcranial magnetic stimulation (TMS) showing that cortical excitability in the M1 of the contralesional hemisphere increases (Liepert et al., 2000b; Shimizu et al., 2002; Butefisch et al., 2003; Manganotti et al., 2008). The degree of imbalance in excitability between hemispheres has been reported to normalize over time in patients with good upper limb recovery (Stinear et al., 2015), while those with poor recovery and more extensive disruption of the ipsilesional corticospinal tract maintain the increased activation in the contralesional hemisphere (Rossini et al., 2003; Ward et al., 2003). However, this is not always the case, as normalization of intracortical inhibition has been reported in patients with poor motor recovery (Liepert et al., 2000b), and the contribution of the contralesional hemisphere may be different in different subsets of patients and at different times post-stroke (Buetefisch, 2015), with a distributed motor network involving both hemispheres contributing to motor recovery (Lotze et al., 2006).

In addition to the uncrossed corticospinal tract, the descending pathways from the contralesional hemisphere to the spinal cord (ipsilateral descending projections) comprise indirect projections, via corticoreticulospinal or corticopropriospinal projections (Ziemann et al., 1999; Alagona et al., 2001). Cortical projections terminating on reticular neurons in the brainstem give rise to the lateral (inhibitory) and medial (excitatory) reticulospinal tracts which have a role in the control of hand function. Following severe damage to the ipsilesional corticospinal tract, the corticoreticulospinal pathway may be the only means of residual control of the paretic upper limb. However, persistent activation of the contralesional hemisphere has been shown to induce excessive activation of ipsilateral reticulospinal projections leading to clinical features of spasticity, including stereotyped limb movement synergies (Li and Francisco, 2015; McPherson et al., 2018) and poor motor recovery (Serrien et al., 2004; Choudhury et al., 2019).

Botulinum toxin type A (BoNT-A) has become a major therapeutic approach for the treatment of focal spasticity. Injected into spastic muscles, it inhibits acetylcholine release from pre-synaptic nerve terminals at the neuromuscular junction, thus weakening the affected muscle (Curra et al., 2004). Although it is generally believed that the clinical benefits of BoNT-A depend primarily on peripheral mechanisms at the level of the muscle, it is possible that such changes may lead to changes in sensorimotor integration within the central nervous system, at spinal cord, brain stem and cortical levels (Curra et al., 2004). A number of studies have identified electrophysiological and functional changes consistent with central nervous system reorganization after intramuscular injection of BoNT-A for movement disorders such as dystonia, in association with clinical improvement (Byrnes et al., 1998; Gilio et al., 2000; Thickbroom et al., 2003; Kojovic et al., 2011). In the case of post-stroke spasticity, BoNT-A injections into spastic muscles led to a reduction of spasticity and fMRI showed an associated reduction in the active motor network and a lateralization of activity to the ipsilesional side (Senkarova et al., 2010; Tomasova et al., 2013). However, the functional changes were reversed once the effects of the injections wore off after 11 weeks (Tomasova et al., 2013).

Paired-pulse TMS can be used to assess the function of intracortical inhibitory and facilitatory circuits in M1 non-invasively in humans (Kujirai et al., 1993; Ziemann et al., 1995, 1996b; Auriat et al., 2015). In this method, a conditioned TMS pulse that is subthreshold or suprathreshold for a motor response activates intracortical circuits and reduces or increases the size of the motor evoked potential (MEP) elicited by a supra-threshold test TMS pulse delivered 3, 10, or 150 ms later. Suppression of the MEP with inter-stimulus intervals of 3 and 150 ms is due to the activation of inhibitory intracortical interneurons and this is known as short interval intracortical inhibition (SICI) and long interval intracortical inhibition (LICI), respectively (Kujirai et al., 1993; Ziemann et al., 1996a; Di Lazzaro et al., 2000, 2005, 2006; Fisher et al., 2002; Ilic et al., 2002; Florian et al., 2008). SICI and LICI are mediated by fast-acting ionotropic gamma-aminobutyric acid A (GABA_*A*_) receptors (Kujirai et al., 1993; Ziemann et al., 1996a, b) and slower-acting metabotropic (GABA_*B*_) receptors (Chen et al., 1998; Siebner et al., 1998; McDonnell et al., 2006), respectively. On the other hand, an increased size of the MEP with inter-stimulus interval of 10 ms is due to the effect of glutaminergic facilitation of corticomotoneuronal (CM) cells (Kujirai et al., 1993; Ziemann et al., 1998; Dyke et al., 2017). Assessing the function of these inhibitory and facilitatory circuits is essential for understanding the neuroplastic changes post-stroke and how these changes might explain functional improvement as well as poor recovery. Previous studies have shown increased intracortical excitability of the M1 area of the contralesional hemisphere in subacute and chronic stroke patients (Liepert et al., 2000a; Shimizu et al., 2002; Butefisch et al., 2003, 2008).

A study using paired pulse TMS to directly investigate changes in cortical excitability in the contralesional hemisphere in chronic stroke survivors showed central changes after BoNT-A injections for upper limb spasticity (Huynh et al., 2013). There was a reduction in SICI in the contralesional hemisphere at baseline which normalized after BoNT-A injections in association with clinical improvements in spasticity. In one patient followed up to 11 weeks post-injection, this effect reverted toward pre-injection levels once the effects of the injection wore off.

The aim of this study was to assess the function of intracortical circuits in the contralesional M1 in chronic stroke patients with disabling upper limb spasticity who received peripheral intramuscular injections of BoNT-A followed by a 12-week upper-limb rehabilitation program.

## Participants

Four participants took part in this study. The criteria for inclusion were unilateral stroke >2 years; age ≥ 18 years; severe unilateral upper limb paresis with a score of 3 or less on the Upper Arm Function and Hand Movements Subscales of the Motor Assessment Scale (MAS); assessed by a rehabilitation physician for potential to benefit from BoNT-A injection to the upper limb for spasticity affecting motor control; no contraindications to BoNT-A injections; able to communicate and understand English; ability and willingness to participate in the study and provide informed consent. We aimed to recruit patients with significant motor dysfunction in the affected upper limb and potentially had persistent changes in cortical reorganization in the contralesional M1 area.

Exclusion criteria were treatment with BoNT-A within the past 3 months; intrathecal baclofen or other anti-spasticity medications; neurolysis or surgery to the affected limb; hypersensitivity to BoNT-A; pregnant or breast feeding; unable to participate in therapy due to cognitive or language impairment; psychiatric or medical illness. Written informed consent was obtained for participation in this study. The study was approved by the Human Research Ethics Committee at Melbourne Health (HREC no. 2018.172).

## Assessments

**FIGURE 1:**
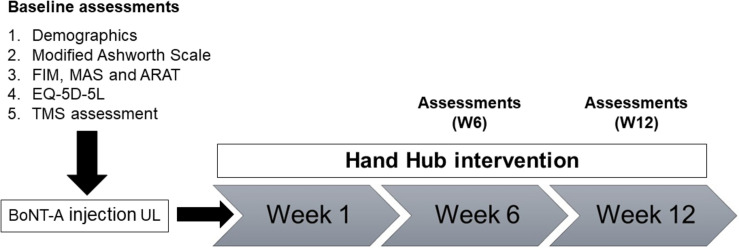
Overview of study design and assessments. MAS, Motor Assessment Scale; ARAT, Action Research Arm Test; FIM, Functional Independence Measure; EQ-5D-5L, Euro-Quality of Life; TMS, transcranial magnetic stimulation.

Assessment time points were baseline before BoNT-A injection, 6-weeks post-BoNT-A injection (W6) and 12 weeks post-BoNT-A injection (W12) (Figure 1).

Clinical assessments were conducted by a rehabilitation physician. Demographic characteristics (age, sex, marital status, education level, employment), and disease-related information (diagnosis, spasticity and other symptoms), medications and co-morbidities were collected. The Functional Independence Measure (FIM) was used to assess dependency (Gosman-Hedstrom and Svensson, 2000), and the Euro-Quality of Life (EQ-5D-5L) questionnaire was used to assess quality of life.

The Upper Arm Function and Hand Movements Subscales of the Motor Assessment Scale (Poole and Whitney, 1988) were used to characterize the severity of impairment of upper limb function, and the Action Research Arm Test (ARAT) (Hsieh et al., 1998) was used to assess functional changes over time. Spasticity in the affected upper limb was evaluated before and after BoNT-A injection using the Modified Ashworth scale (Li et al., 2014) (0 = no increase in muscle tone to 4 = rigid flexion or extension). A score of 1 + was assigned the value of 1.5.

All participants were screened at baseline using the TMS Adult Safety Screen (Keel et al., 2001) to ensure there would be no contraindications for TMS. Short- and long-interval cortical inhibition (SICI and LICI) and intracortical facilitation (ICF) were measured with paired-pulse TMS (Magstim Bistim^2^) at baseline, W6, and W12. Participants were seated upright in a comfortable chair with their hands and forearms supported on a pillow. Paired-pulse TMS was delivered with two Magstim 200 stimulators (Magstim Company Limited, United Kingdom) connected with a Bistim module (Magstim Company Limited, United Kingdom). As a result, the pulses were delivered through one figure of eight coil (70 mm). The coil was placed over the contralesional M1 area with an angle of 45° to the midline and tangential to the scalp. This coil orientation induces current flow in a posterior to anterior direction in the M1 area. The optimal site for evoking a MEP in the contralateral first dorsal interosseous (FDI) muscle was marked as the “hot spot.” Throughout each experiment, the position and orientation of the coil were monitored regularly to ensure that the same area was stimulated with each stimulus. Resting motor threshold (RMT) for inducing MEP in the targeted FDI was measured in steps of 2% maximum stimulator output, and defined as the lowest intensity for inducing 3 out of 5 successive MEPs that exceed 50 μV peak-to-peak amplitude (Rossini et al., 1994).

The MEPs were recorded from FDI muscle on the unaffected hand using pre-gelled self-adhesive bipolar Ag/AgCl disposable surfaces electrodes (3 cm inter-electrode distance). To reduce the skin resistance, skin under the electrodes was cleaned with an alcohol swab and the electrodes were secured with non-allergenic medical tape. The ground electrode was placed ipsilaterally on the styloid process of the ulna. The FDI location was determined based on anatomical landmarks (Perotto and Delagi, 2005) and by observing the muscle contraction during index finger abduction. Then, the EMG activity of the FDI was monitored online during maximum contraction to confirm the accuracy of the EMG electrode placement.

The raw EMG signals were filtered (10–500 Hz), amplified (×1000) and sampled at 2000 Hz, and recorded by a laboratory analog-digital interface (PowerLab 8/30, ADInstruments, Australia). The unconditioned test TMS intensity was adjusted to produce a test MEP in FDI at rest of about 1 mV amplitude. Conditioning TMS intensity was adjusted to 0.8 × RMT for each participant with inter-stimulus intervals (ISI) of 3, 10, or 150 ms. Single or paired-pulse TMS was delivered in blocks of 20 stimuli (10 s interval between stimuli) with each ISI. MEP responses were quantified off-line from the digitized averages of rectified MEPs. The peak-to-peak amplitude of MEP responses and the area of the conditioned and unconditioned MEPs were measured using a custom-designed macro in PowerLab 8/30 software. The size of the conditioned MEPs was expressed as a percentage of the unconditioned test MEPs in order to quantify SICI, LICI, and ICF.

Clinical and electrophysiological assessments were performed on patients within 3 days prior to BoNT-A injection and repeated at W6 when the clinical effects were maximal, and again at W12. BoNT-A was injected into the affected forearm flexor muscles, including the flexor carpi radialis (FCR), flexor carpi ulnaris (FCU), and flexor digitorum profundus (FDP), at a dose determined by the treating physician, depending on the severity of the spasticity.

Following BoNT-A injections, as per routine care, participants were referred to an ambulatory rehabilitation program in the ‘Hand Hub’ at a rehabilitation hospital. The Hand Hub incorporates inexpensive devices, such as the AbleX and AbleM (Im-Able Ltd., New Zealand), and the ReJoyce (Rehabtronics Inc.), which provide a range of computer-controlled exercises, catering for patients with varying levels of severity of arm and hand impairment. Patients were asked to attend the Hand Hub twice per week for 12 weeks.

## Results

Demographic, sociodemographic and clinical details of the participants at baseline are shown in Table 1.

**TABLE 1:** Demographic and clinical characteristics.

**Variables**	**Participant 1**	**Participant 2**	**Participant 3**	**Participant 4**
Age (years)	65.4	69.3	36.5	69.6
Gender	Male	Female	Female	Male
Ethnicity	Caucasian	Caucasian	Caucasian	Caucasian
Marital status	Married	Married	Divorced	Single
Employment	Unemployed	Unemployed	Unemployed	Unemployed
Carer	No	Yes	Yes	No
Stroke type	Hemorrhagic	Ischemic	Hemorrhagic	Ischemic
Location	Basal ganglia	MCA	Basal ganglia	MCA
Disease duration (years)	6.9	8.9	17.3	3.1
Affected side	Left	Left	Left	Left
Hand dominance	Right	Right	Right	Left
Comorbidities	HtN, DM	Htn	None	Htn
Cognitive impairment	No	No	No	No
Sensory-perceptual deficit	No	Yes	No	No
Speech/communication issues	No	No	No	No
Pain	No	Mild	Mild	No
Fatigue	Moderate	Mild	Mild	Moderate
BoNT-A (Xeomin) total injected dose	200 units	300 units	200 units	400 units
UL muscle injected	FDS, FCU, FCR	FDS, FDP, FPL	FDS, FDP, FCR	FDS, FDP, FPL, biceps

*DVT, deep vein thrombosis; BoNT-A, botulinum toxin-A; DM, diabetes mellitus; FDS, flexor digitorum superficialis; FCU, flexor carpi ulnaris; FCR, flexor carpi radialis; FDP, flexor digitorum profundus; FPL, flexor pollicis longus; Htn, hypertension; PE, pulmonary embolism.*

Three participants attended the Hand Hub twice per week for 12 weeks, as instructed; however, P3 was only able to attend once per week for 12 weeks. There were no significant changes in the participants’ FIM scores at any assessment time points, indicating minimal functional improvement. Improvement in UL functions in ARAT ‘Gross’ subscale was only noted for P1 at W6 which was maintained till W12. Modified Ashworth Scale score changes for different injected muscles varied amongst participants at both W6 and W12 assessments (Please refer to Table 2 for detailed scores). All participants reported improvement in their health status in EQ-5D ‘overall health’ subscale at both W6 and W12 assessments (Table 2).

**TABLE 2:** Total scores of measurement scales over time.

**Scales**	**Participant 1**			**Participant 2**			**Participant 3**			**Participant 4**		
	**B**	**W6**	**W12**	**B**	**W6**	**W12**	**B**	**W6**	**W12**	**B**	**W6**	**W12**
**ARAT**												
Grasp (0–18)	0	0	0	0	0	0	0	0	0	0	0	0
Grip (0–12)	0	0	0	0	0	0	0	0	0	0	0	0
Pinch (0–18)	0	0	0	0	0	0	0	0	0	0	0	0
Gross movement (0–9)	4	9	9	2	2	3	2	3	3	2	3	3
Total (0–57)	4	9	9	2	2	3	2	3	3	2	3	3
**FIM Motor**												
Total (13–91)	74	75	75	63	63	63	74	79	78	74	79	79
Self-care (6.42)	31	31	31	27	27	27	30	32	33	32	35	35
Sphincter (2–14)	14	14	14	12	12	12	14	14	14	12	14	14
Locomotion (2–14)	11	12	12	7	7	7	9	12	12	12	12	12
Mobility (3-21)	18	18	18	17	17	17	21	21	19	18	18	18
**FIM Cognition**												
Total (5–35)	33	33	33	30	30	30	30	30	30	35	35	35
Communication (2–14)	12	12	12	12	12	12	12	12	12	14	14	14
Psychosocial (1–7)	7	7	7	6	6	6	6	6	6	7	7	7
Cognition (2–14)	14	14	14	12	12	12	12	12	12	14	14	14
**Motor Assessment Scale**												
Upper arm movement (0–6)	2	2	2	2	3	4	0	0	1	1	1	1
Hand movement (0–6)	1	2	2	1	2	2	0	0	1	1	1	1
Advanced hand activities (0–6)	1	1	1	0	0	0	0	0	0	0	0	0
Total (0–18)	4	5	5	3	5	6	0	0	2	2	2	2
**Modified Ashworth Scale (0–4)**												
FCR	3	2	3				3	2	2			
FCU	3	2	2									
FDS	3	2	2	3	2	3	3	2	2	3	3	3
FDP				3	3	3	3	2	3	3	3	3
FPL				3	3	3				3	3	3
Biceps										2	+ 1	+1
**EQ-5D**												
Mobility (1–5)	2	2	1	3	1	1	2	3	3	1	1	1
Self-care (1–5)	2	2	2	3	3	3	2	1	1	1	1	1
Daily activity (1–5)	2	3	2	3	4	3	2	1	1	0	0	0
Pain/discomfort (1–5)	1	0	1	3	2	1	2	2	2	0	1	0
Anxiety/depression (1–5)	1	0	1	3	0	0	2	2	2	0	1	0
Overall health (0–100)	70	90	75	70	70	80	70	70	75	60	80	85

*B, Baseline assessment; W6, week 6 assessment; W12, Week 12 assessment; ARAT, Action Research Arm Test; EQ-5D-5L: Euro-Quality of Life; FIM, Functional Independence Measure; FDS, Flexor digitorum superficialis; FCU, flexor carpi ulnaris; FCR, flexor carpi radialis; FDP, flexor digitorum profundus; FPL, flexor pollicis longus.*

**FIGURE 2:**
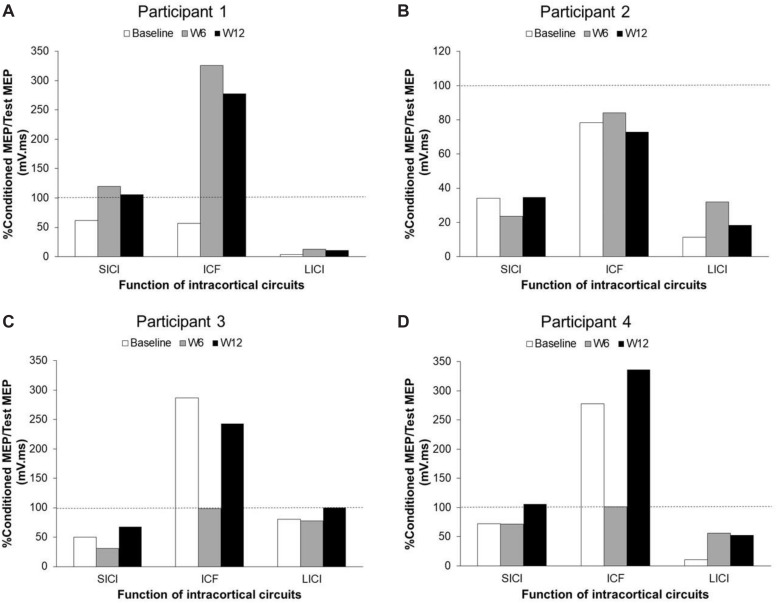
Changes of SICI, LICI, and ICF in unaffected M1 areas in each participant with chronic stroke after BoNT-A injection and 6 weeks upper-limb rehabilitation program. Panel **(A)** shows participant 1 data. Panel **(B)** shows participant 2 data. Panel **(C)** shows participant 3 data. Panel **(D)** shows participant 4 data. SICI, short-interval intracortical inhibition; LICI, long-interval intracortical inhibition; ICF, intracortical facilitation; MEP, motor-evoked potential; W, week.

The TMS parameters used for each participant are presented in Table 3. Figure 2 presents the changes in SICI, LICI and ICF at three time points (baseline, W6 and W12) in each participant (Figures 2A–D). Panel A shows the levels of SICI and LICI and ICF for P1. As it can be seen, LICI level remained at the similar level for this participant throughout the study (3.5–13%); however, SICI was completely abolished at W6 (120%) and W12 (106%). On the other hand, ICF was higher at W6 (325%) and W12 (278%). P2 did not show any ICF throughout the study (73%–84%) (Panel B). This participant showed some level of SICI (34%) and LICI (11%) at baseline with slight changes at W6 (SICI: 24%; LICI: 32%) and 12 (SICI: 35%; LICI: 18%). P3 showed some level of SICI at baseline (50%) with slight changes at W6 (32%) and 12 (67%). This participant also showed some level of ICF at baseline (287%); however, it was completely abolished at W6 (98%) and then fully recovered at W12 (243%). This participant showed some level of LICI at baseline (81%) with no change at W6 (78%). However, it was completely abolished at W12 (100%). The change in ICF for P4 was very similar to P3 (Baseline: 278%; W6: 101%; W12: 336%). However, this participant showed some level of LICI at baseline (11%) which was reduced at W6 (56%) and W12 (53%). This participant also showed some level of SICI at baseline (72%) and W6 (72%) which was completely abolished at W12 (106%).

**TABLE 3:** TMS parameters used for each patient in each session.

**Participant**	**Resting motor threshold**			**Conditioning intensity**			**Test intensity**		
	**(RMT, % Magstim output)**			**(RMT × 0.8)**			**(% Magstim output to produce 1 mV MEPs)**		
	**Session 1**	**Session 2**	**Session 3**	**Session 1**	**Session 2**	**Session 3**	**Session 1**	**Session 2**	**Session 3**
P1	50%	39%	50%	40%	31%	40%	62%	63%	59%
P2	58%	58%	53%	46%	46%	42%	70%	70%	70%
P3	63%	61%	51%	50%	49%	41%	69%	66%	60%
P4	61%	48%	50%	49%	38%	40%	70%	58%	60%

## Discussion

In this study, we assessed the intracortical inhibitory and facilitatory circuits in the contralesional M1 area in patients with chronic stroke after BoNT-A injection and completing 12 weeks of upper-limb rehabilitation. The level of SICI, LICI, and ICF provided an indication of the function of these circuits in contralesional M1 area. Four patients participated in this study. Three can be considered as “old” adults (≥65 years) and one as a young adult (P3, 36 years). The age-related changes in the level of SICI, LICI, and ICF are not clear. It has been shown that the level of SICI at rest does not change with age (Oliviero et al., 2006; Smith et al., 2009); however, the level of LICI is reduced (Opie and Semmler, 2014). Another study showed increased SICI and LICI and less ICF in older adults compared to young adults at rest (McGinley et al., 2010). One possible reason behind the discrepancy among these reported values could be the selected TMS parameters in each study (e.g., conditioning intensity). In the current study, we did not include any age-matched neurologically intact participants; therefore, we are not able to provide any comment on the possible physiological age-related changes to SICI, LICI, or ICF levels before stroke or the TMS used parameters in this study compared to the previous studies. However, it is important to consider the possible contribution of age-related changes in these circuits prior to the stroke.

Similar discrepancies can also be seen in studies in the stroke population. Some studies showed a reduction in SICI levels in the contralesional hemisphere after stroke (Shimizu et al., 2002; Butefisch et al., 2003) which normalized gradually over 4 months post-stroke (Shimizu et al., 2002). However, another study showed no changes in SICI level and an increase in ICF level in contralesional hemisphere after stroke (Wittenberg et al., 2007). In addition, Conforto et al. (2008) reported increased levels of SICI in the contralesional hemisphere in chronic stroke patients (Conforto et al., 2008). In the current study, the level of SICI and LICI were similar at baseline for the older (P1, P2, and P4) and young (P3) participants, despite differences in the type and location of stroke. However, the ICF level was different at baseline among these patients. P1 and P2 did not show any level of ICF at baseline but similar levels of ICF were recorded in the other two participants (P3 and P4) despite the difference in age. Edwards et al. (2013) assessed changes in the onset thresholds for SICI and ICF in stroke patients compared to age-matched healthy adults in both ipsi- and contralesional hemisphere (Edwards et al., 2013) and reported significant reductions in onset thresholds for SICI in the contralesional hemisphere and significant reductions in onset thresholds for ICF in the ipsilesional hemisphere in patients with chronic stroke (Edwards et al., 2013). One possible reason that ICF was not recorded for P1 and P2 could be because of changes in the onset threshold for ICF in these patients and the possibility that the stimulus intensity used in this study was insufficient to activate the facilitatory networks in these patients. Further studies are needed to investigate this issue with larger numbers of participants.

After BoNT-A injection and 12 weeks of rehabilitation, there was little change in spasticity in the injected muscles. While the MAS is very commonly used for the assessment of spasticity, it lacks precision with respect to distinguishing between neural and non-neural aspects of increased muscle tone (Pandyan et al., 1999). Peripheral changes, such as muscle contracture, may have confounded the measurement in our study participants with chronic stroke, and use of the Tardieu Scale, which can distinguish between dynamic and non-dynamic components of increased muscle tone, may have been more appropriate (Patrick and Ada, 2006). Changes in upper limb function following BoNT-A injections were different for each participant, with only P1 showing improvement in gross motor function. Interestingly, despite the lack of functional improvement, all participants reported an improvement in quality of life, in contrast with other studies which have reported moderate improvements in upper limb function following botulinum toxin injections for spasticity in stroke survivors (Foley et al., 2013), but no improvement in quality of life (Caty et al., 2009; McCrory et al., 2009). The lack of functional improvement may have resulted from the relatively low intensity of upper limb rehabilitation provided post-injection. Ideally, a higher intensity of rehabilitation could potentially maximize the potential for functional change through a window for enhanced brain plasticity occasioned by the BoNT-A injections (Curra et al., 2004), although it has been noted that BoNT-A injections may be of greater benefit in improving passive upper limb functions (care of the affected limb) and these would not be picked up by measures of active function (Foley et al., 2013).

Each participant showed a different pattern of changes in the levels of SICI, LICI, and ICF (Figure 2). Huynh et al. (2013) reported that the decreased level of SICI in the contralesional hemisphere in chronic stroke patients was normalized after BoNT-A injection in spastic hand muscles and returned to pre-injection levels when the effect of BoNT-A wore off (Huynh et al., 2013). They suggested that the decreased level of SICI is the possible mechanism for development of spasticity on the affected side (Huynh et al., 2013). In the present study, none of the participants showed a decrease in the level of SICI before the BoNT-A injection. After injection, the level of SICI did not change for two participants (P2 and P3) throughout the study; however, it was completely abolished for P1 at W6 and W12, and for P4 at W12.

One possible reason for these observations is the effect of BoNT-A on activation thresholds for the intracortical circuits. It has been suggested that BoNT-A might affect both hemispheres by spreading through the blood–brain barrier or via retrograde axonal transport through motor and propriospinal pathways (Huynh et al., 2013). Both ipsilateral and contralateral corticospinal tracts are involved in controlling upper limb function post-stroke (Ziemann et al., 1999; Ward et al., 2003). The participants in this study had a significant degree of spasticity which did not decrease after a single BoNT-A injection, contrary to the findings of other studies (Huynh et al., 2013; Tomasova et al., 2013). This observation suggests that the BoNT-A dosage in this study may not have been optimal. It has been shown that in some cases, several injections are needed to significantly decrease the severity of spasticity in the targeted muscles (Kaku and Simpson, 2016). Therefore, it is possible that the dose of BoNT-A used in this study may have been insufficient to have a direct effect on the activation threshold of the intracortical circuits. Future studies could consider increasing the follow up period to 6–12 months, which might include multiple treatments with BoNT-A in order to properly evaluate the effect of BoNT-A on intracortical circuits in contralesional M1 area. Participants in this study also showed very poor recovery of upper limb function, which suggests the persistent involvement of ipsilateral descending tracts in upper limb function in these patients (Jang, 2007). Therefore, it may require further doses of BoNT-A and a more intensive period of rehabilitation to alter the sensory stimuli from the spastic muscles and change the reorganization of the motor areas in both hemispheres and in turn affect the activation threshold for intracortical inhibitory circuits in M1 areas on both sides.

In paired-pulse TMS technique, the intensity of the conditioning stimulus is determined based on the MEP threshold, which is the lowest intensity that can produce MEPs ≥ 50 μV peak-to-peak amplitude at rest (Rossini et al., 1994, 2015). The relationship between this intensity and the level of inhibition (SICI) is a U-shaped curve (Chen et al., 1998; Ilic et al., 2002). Kujirai et al. (1993) showed that the inhibitory effect becomes evident with a low conditioning intensity around 60% of RMT and ISI of 3 ms, reaches the maximum level with 80% of RMT, and is reversed with a higher conditioning intensity. They proposed that the decreased level of inhibition with higher conditioning intensity is probably due to the recruitment of facilitatory circuits (Kujirai et al., 1993; Peurala et al., 2008). It has been shown that facilitatory circuits have a higher threshold compared to inhibitory circuits (Davey et al., 1994; Ziemann et al., 1996b; Awiszus et al., 1999; Ilic et al., 2002) and are evident only if the conditioning intensity is ≥0.8 × RMT (Ilic et al., 2002). In this study, the conditioning intensity was 80% of RMT and the ISI was 3 ms. Use of these parameters in P2 and P3 could still demonstrate a significant level of SICI at all three assessment points, and BoNT-A injection and upper limb exercises did not change the threshold of the intracortical circuits in the contralesional M1 area in these patients. However, the same parameters significantly affected the threshold of these intracortical interneurons in P1, such that 80% of RMT was strong enough to stimulate the facilitatory circuits and completely overcome the inhibitory effect on CM cells.

We were not able to assess whether these observations reflected the changes in the level of spasticity or functional improvement in patients. FIM has well-known floor and ceiling effects, which were evident in this study which showed minimal improvement in FIM scores. Further, we did not assess the direct effects of the BONT-A for any of the secondary outcomes. This was beyond the aim of this study. Understanding how inhibitory and facilitatory circuits in the contralesional hemisphere are affected by BoNT-A injections, the severity of spasticity, and functional improvement warrants further investigation.

## Conclusion

Despite similar clinical presentations, the four participants in this study showed a mixed pattern of levels of intracortical inhibition and excitation in the contralesional M1 at baseline and responses to BoNT-A injections and follow-up rehabilitation. This demonstrates variability in the type and degree of cortical reorganization associated with severe spasticity and its treatment after stroke, and further research is necessary to clarify this issue.

Future research should therefore consider the following issues in the study design: including a larger number of participants; including age-matched neurologically intact adults; optimizing the BoNT-A dosage for participants, increasing the follow-up duration to 6 or 12 months; assessing spinal excitability as well as cortical excitability to be able to comment on the level of contributions at both spinal and cortical levels.

## Data Availability Statement

The raw data supporting the conclusions of this article will be made available by the authors, without undue reservation.

## Ethics Statement

The study was approved by the Melbourne Health Human Research Ethics Committee (HREC no. 2018.172). Written informed consent was obtained from all participants for participation in this study and also for the publication of any potentially identifiable images or data from this study.

## Author Contributions

MG designed the study and obtained the ethics approval. PH completed the BoNT-A injections. PH and MZ collected the TMS data. MZ completed the data reduction. All authors contributed to the interpretation of the results and manuscript writing.

## Conflict of Interest

The authors declare that the research was conducted in the absence of any commercial or financial relationships that could be construed as a potential conflict of interest.

## References

[B1] Alagona G. Delvaux V. Gerard P. De Pasqua V. Pennisi G. Delwaide P. J. (2001). Ipsilateral motor responses to focal transcranial magnetic stimulation in healthy subjects and acute-stroke patients. *Stroke* 32 1304–1309. 10.1161/01.str.32.6.1304 11387491

[B2] Auriat A. M. Neva J. L. Peters S. Ferris J. K. Boyd L. A. (2015). A review of transcranial magnetic stimulation and multimodal neuroimaging to characterize post-stroke neuroplasticity. *Front. Neurol.* 6:226. 10.3389/fneur.2015.00226 26579069 PMC4625082

[B3] Awiszus F. Feistner H. Urbach D. Bostock H. (1999). Characterisation of paired-pulse transcranial magnetic stimulation conditions yielding intracortical inhibition or I-wave facilitation using a threshold-hunting paradigm. *Exp. Brain Res.* 129 317–324. 10.1007/s002210050901 10591905

[B4] Buetefisch C. M. (2015). Role of the contralesional hemisphere in post-stroke recovery of upper extremity motor function. *Front. Neurol.* 6:214. 10.3389/fneur.2015.00214 26528236 PMC4607877

[B5] Butefisch C. M. Netz J. Wessling M. Seitz R. J. Homberg V. (2003). Remote changes in cortical excitability after stroke. *Brain* 126 (Pt 2), 470–481. 10.1093/brain/awg044 12538413

[B6] Butefisch C. M. Wessling M. Netz J. Seitz R. J. Homberg V. (2008). Relationship between interhemispheric inhibition and motor cortex excitability in subacute stroke patients. *Neurorehabil. Neural Repair* 22 4–21. 10.1177/1545968307301769 17507644

[B7] Byrnes M. L. Thickbroom G. W. Wilson S. A. Sacco P. Shipman J. M. Stell R. (1998). The corticomotor representation of upper limb muscles in writer’s cramp and changes following botulinum toxin injection. *Brain* 121 (Pt 5), 977–988. 10.1093/brain/121.5.977 9619198

[B8] Caty G. D. Detrembleur C. Bleyenheuft C. Deltombe T. Lejeune T. M. (2009). Effect of upper limb botulinum toxin injections on impairment, activity, participation, and quality of life among stroke Patients. *Stroke* 40 2589–2591. 10.1161/strokeaha.108.544346 19407231

[B9] Chen R. Tam A. Butefisch C. Corwell B. Ziemann U. Rothwell J. C. (1998). Intracortical inhibition and facilitation in different representations of the human motor cortex. *J. Neurophysiol.* 80 2870–2881. 10.1152/jn.1998.80.6.2870 9862891

[B10] Choudhury S. Shobhana A. Singh R. Sen D. Anand S. S. Shubham S. (2019). The relationship between enhanced reticulospinal outflow and upper limb function in chronic stroke patients. *Neurorehabil. Neural Repair* 33 375–383. 10.1177/1545968319836233 30913964

[B11] Conforto A. B. Santos R. L. Farias S. N. Marie S. K. Mangini N. Cohen L. G. (2008). Effects of somatosensory stimulation on the excitability of the unaffected hemisphere in chronic stroke patients. *Clinics* 63 735–740. 10.1590/s1807-59322008000600005 19060993 PMC2664271

[B12] Curra A. Trompetto C. Abbruzzese G. Berardelli A. (2004). Central effects of botulinum toxin type a: evidence and supposition. *Mov. Disord.* 19(Suppl. 8), S60–S64. 15027056 10.1002/mds.20011

[B13] Davey N. J. Romaiguere P. Maskill D. W. Ellaway P. H. (1994). Suppression of voluntary motor activity revealed using transcranial magnetic stimulation of the motor cortex in man. *J. Physiol.* 477 (Pt 2), 223–235. 10.1113/jphysiol.1994.sp020186 7932215 PMC1155624

[B14] Di Lazzaro V. Oliviero A. Meglio M. Cioni B. Tamburrini G. Tonali P. (2000). Direct demonstration of the effect of lorazepam on the excitability of the human motor cortex. *Clin. Neurophysiol.* 111 794–799. 10.1016/s1388-2457(99)00314-4 10802448

[B15] Di Lazzaro V. Pilato F. Dileone M. Ranieri F. Ricci V. Profice P. (2006). GABAA receptor subtype specific enhancement of inhibition in human motor cortex. *J. Physiol.* 575 (Pt 3), 721–726. 10.1113/jphysiol.2006.114694 16809358 PMC1995685

[B16] Di Lazzaro V. Pilato F. Dileone M. Tonali P. A. Ziemann U. (2005). Dissociated effects of diazepam and lorazepam on short-latency afferent inhibition. *J. Physiol. Lond.* 569 315–323. 10.1113/jphysiol.2005.092155 16141274 PMC1464195

[B17] Dyke K. Pepes S. E. Chen C. Kim S. Sigurdsson H. P. Draper A. (2017). Comparing GABA-dependent physiological measures of inhibition with proton magnetic resonance spectroscopy measurement of GABA using ultra-high-field MRI. *Neuroimage* 152 360–370. 10.1016/j.neuroimage.2017.03.011 28284797 PMC5440178

[B18] Edwards J. D. Meehan S. K. Linsdell M. A. Borich M. R. Anbarani K. Jones P. W. (2013). Changes in thresholds for intracortical excitability in chronic stroke: more than just altered intracortical inhibition. *Restor. Neurol. Neurosci.* 31 693–705. 10.3233/rnn-120300 23963339

[B19] Fisher R. J. Nakamura Y. Bestmann S. Rothwell J. C. Bostock H. (2002). Two phases of intracortical inhibition revealed by transcranial magnetic threshold tracking. *Exp. Brain Res.* 143 240–248. 10.1007/s00221-001-0988-2 11880900

[B20] Florian J. Muller -Dahlhaus M. Liu Y. Ziemann U. (2008). Inhibitory circuits and the nature of their interactions in the human motor cortex a pharmacological TMS study. *J. Physiol.* 586 495–514. 10.1113/jphysiol.2007.142059 17991698 PMC2375584

[B21] Foley N. Pereira S. Salter K. Fernandez M. M. Speechley M. Sequeira K. (2013). Treatment with botulinum toxin improves upper-extremity function post stroke: a systematic review and meta-analysis. *Arch. Phys. Med. Rehabil.* 94 977–989. 10.1016/j.apmr.2012.12.006 23262381

[B22] Gilio F. Curra A. Lorenzano C. Modugno N. Manfredi M. Berardelli A. (2000). Effects of botulinum toxin type A on intracortical inhibition in patients with dystonia. *Ann. Neurol.* 48 20–26. 10.1002/1531-8249(200007)48:1<20::aid-ana5>3.0.co;2-u 10894212

[B23] Gosman-Hedstrom G. Svensson E. (2000). Parallel reliability of the functional independence measure and the Barthel ADL index. *Disabil. Rehabil.* 22 702–715. 10.1080/09638280050191972 11117590

[B24] Grefkes C. Fink G. R. (2011). Reorganization of cerebral networks after stroke: new insights from neuroimaging with connectivity approaches. *Brain* 134 1264–1276. 10.1093/brain/awr033 21414995 PMC3097886

[B25] Hsieh C. L. Hsueh I. P. Chiang F. M. Lin P. H. (1998). Inter-rater reliability and validity of the action research arm test in stroke patients. *Age Ageing* 27 107–113. 10.1093/ageing/27.2.107 16296669

[B26] Huynh W. Krishnan A. V. Lin C. S. Vucic S. Katrak P. Hornberger M. (2013). Botulinum toxin modulates cortical maladaptation in post-stroke spasticity. *Muscle Nerve* 48 93–99. 10.1002/mus.23719 23625819

[B27] Ilic T. V. Meintzschel F. Cleff U. Ruge D. Kessler K. R. Ziemann U. (2002). Short-interval paired-pulse inhibition and facilitation of human motor cortex: the dimension of stimulus intensity. *J. Physiol.* 545 (Pt 1), 153–167. 10.1113/jphysiol.2002.030122 12433957 PMC2290644

[B28] Jang S. H. (2007). A review of motor recovery mechanisms in patients with stroke. *Neurorehabilitation* 22 253–259. 10.3233/nre-2007-22401 17971614

[B29] Kaku M. Simpson D. M. (2016). Spotlight on botulinum toxin and its potential in the treatment of stroke-related spasticity. *Drug Des. Dev. Ther.* 10 1085–1099. 10.2147/DDDT.S80804 PMC4789850 27022247

[B30] Keel J. C. Smith M. J. Wassermann E. M. (2001). A safety screening questionnaire for transcranial magnetic stimulation. *Clin. Neurophysiol.* 112:720 10.1016/s1388-2457(00)00518-6 11332408

[B31] Kojovic M. Caronni A. Bologna M. Rothwell J. C. Bhatia K. P. Edwards M. J. (2011). Botulinum toxin injections reduce associative plasticity in patients with primary dystonia. *Mov. Disord.* 26 1282–1289. 10.1002/mds.23681 21469207 PMC4235250

[B32] Kujirai T. Caramia M. D. Rothwell J. C. Day B. L. Thompson P. D. Ferbert A. (1993). Corticocortical inhibition in human motor cortex. *J. Physiol.* 471 501–519. 10.1113/jphysiol.1993.sp019912 8120818 PMC1143973

[B33] Li F. Wu Y. Li X. (2014). Test-retest reliability and inter-rater reliability of the modified tardieu scale and the modified ashworth scale in hemiplegic patients with stroke. *Eur. J. Phys. Rehabil. Med.* 50 9–15. 24309501

[B34] Li S. Francisco G. E. (2015). New insights into the pathophysiology of post-stroke spasticity. *Front. Hum. Neurosci.* 9:192. 10.3389/fnhum.2015.00192 25914638 PMC4392691

[B35] Liepert J. Hamzei F. Weiller C. (2000a). Motor cortex disinhibition of the unaffected hemisphere after acute stroke. *Muscle Nerve* 23 1761–1763. 10.1002/1097-4598(200011)23:11<1761::aid-mus14>3.0.co;2-m 11054757

[B36] Liepert J. Storch P. Fritsch A. Weiller C. (2000b). Motor cortex disinhibition in acute stroke. *Clin. Neurophysiol.* 111 671–676. 10.1016/s1388-2457(99)00312-0 10727918

[B37] Lotze M. Markert J. Sauseng P. Hoppe J. Plewnia C. Gerloff C. (2006). The role of multiple contralesional motor areas for complex hand movements after internal capsular lesion. *J. Neurosci.* 26 6096–6102. 10.1523/jneurosci.4564-05.2006 16738254 PMC6675223

[B38] Manganotti P. Acler M. Zanette G. P. Smania N. Fiaschi A. (2008). Motor cortical disinhibition during early and late recovery after stroke. *Neurorehabil. Neural Repair* 22 396–403. 10.1177/1545968307313505 18326890

[B39] McCrory P. Turner-Stokes L. Baguley I. J. De Graaff S. Katrak P. Sandanam J. (2009). Botulinum toxin A for treatment of upper limb spasticity following stroke: a multi-centre randomized placebo-controlled study of the effects on quality of life and other person-centred outcomes. *J. Rehabil. Med.* 41 536–544. 10.2340/16501977-0366 19543664

[B40] McDonnell M. N. Orekhov Y. Ziemann U. (2006). The role of GABA(B) receptors in intracortical inhibition in the human motor cortex. *Exp. Brain Res.* 173 86–93. 10.1007/s00221-006-0365-2 16489434

[B41] McGinley M. Hoffman R. L. Russ D. W. Thomas J. S. Clark B. C. (2010). Older adults exhibit more intracortical inhibition and less intracortical facilitation than young adults. *Exp. Gerontol.* 45 671–678. 10.1016/j.exger.2010.04.005 20417265 PMC2926152

[B42] McPherson J. G. Chen A. Ellis M. D. Yao J. Heckman C. J. Dewald J. P. A. (2018). Progressive recruitment of contralesional cortico-reticulospinal pathways drives motor impairment post stroke. *J. Physiol.* 596 1211–1225. 10.1113/jp274968 29457651 PMC5878212

[B43] Oliviero A. Profice P. Tonali P. A. Pilato F. Saturno E. Dileone M. (2006). Effects of aging on motor cortex excitability. *Neurosci. Res.* 55 74–77. 10.1016/j.neures.2006.02.002 16584795

[B44] Opie G. M. Semmler J. G. (2014). Age-related differences in short- and long-interval intracortical inhibition in a human hand muscle. *Brain Stimul.* 7 665–672. 10.1016/j.brs.2014.06.014 25088463

[B45] Pandyan A. D. Johnson G. R. Price C. I. M. Curless R. H. Barnes M. P. Rodgers H. (1999). A review of the properties and limitations of the Ashworth and modified Ashworth Scales as measures of spasticity. *Clin. Rehabil.* 13 373–383. 10.1191/026921599677595404 10498344

[B46] Pascual-Leone A. Amedi A. Fregni F. Merabet L. B. (2005). The plastic human brain cortex. *Annu. Rev. Neurosci.* 28 377–401. 16022601 10.1146/annurev.neuro.27.070203.144216

[B47] Patrick E. Ada L. (2006). The Tardieu Scale differentiates contracture from spasticity whereas the Ashworth Scale is confounded by it. *Clin. Rehabil.* 20 173–182. 10.1191/0269215506cr922oa 16541938

[B48] Perotto A. Delagi E. F. (2005). *Anatomical Guide for the Electromyographer: The Limbs and Trunk.* Springfield, IL: Charles C Thomas Publisher Ltd.

[B49] Peurala S. H. Muller-Dahlhaus J. F. Arai N. Ziemann U. (2008). Interference of short-interval intracortical inhibition (SICI) and short-interval intracortical facilitation (SICF). *Clin. Neurophysiol.* 119 2291–2297. 10.1016/j.clinph.2008.05.031 18723394

[B50] Poole J. L. Whitney S. L. (1988). Motor assessment scale for stroke patients: concurrent validity and interrater reliability. *Arch. Phys. Med. Rehabil.* 69(3 Pt 1), 195–197. 3348720

[B51] Rossini P. M. Barker A. T. Berardelli A. Caramia M. D. Caruso G. Cracco R. Q. (1994). Non-invasive electrical and magnetic stimulation of the brain, spinal cord and roots: basic principles and procedures for routine clinical application. Report of an IFCN committee. *Electroencephalogr. Clin. Neurophysiol.* 91 79–92. 10.1016/0013-4694(94)90029-9 7519144

[B52] Rossini P. M. Burke D. Chen R. Cohen L. G. Daskalakis Z. Di Iorio R. (2015). Non-invasive electrical and magnetic stimulation of the brain, spinal cord, roots and peripheral nerves: basic principles and procedures for routine clinical and research application. An updated report from an I.F.C. Committee. *Clin. Neurophysiol.* 126 1071–1107. 10.1016/j.clinph.2015.02.001 25797650 PMC6350257

[B53] Rossini P. M. Calautti C. Pauri F. Baron J. C. (2003). Post-stroke plastic reorganisation in the adult brain. *Lancet Neurol.* 2 493–502. 10.1016/s1474-4422(03)00485-x 12878437

[B54] Senkarova Z. Hlustik P. Otruba P. Herzig R. Kanovsky P. (2010). Modulation of cortical activity in patients suffering from upper arm spasticity following stroke and treated with botulinum toxin A: an fMRI study. *J. Neuroimaging* 20 9–15. 10.1111/j.1552-6569.2009.00375.x 19453837

[B55] Serrien D. J. Strens L. H. Cassidy M. J. Thompson A. J. Brown P. (2004). Functional significance of the ipsilateral hemisphere during movement of the affected hand after stroke. *Exp. Neurol.* 190 425–432. 10.1016/j.expneurol.2004.08.004 15530881

[B56] Shimizu T. Hosaki A. Hino T. Sato M. Komori T. Hirai S. (2002). Motor cortical disinhibition in the unaffected hemisphere after unilateral cortical stroke. *Brain* 125 (Pt 8), 1896–1907. 10.1093/brain/awf183 12135979

[B57] Siebner H. R. Dressnandt J. Auer C. Conrad B. (1998). Continuous intrathecal baclofen infusions induced a marked increase of the transcranially evoked silent period in a patient with generalized dystonia. *Muscle Nerve* 21 1209–1212. 10.1002/(sici)1097-4598(199809)21:9<1209::aid-mus15>3.0.co;2-m 9703450

[B58] Smith A. E. Ridding M. C. Higgins R. D. Wittert G. A. Pitcher J. B. (2009). Age-related changes in short-latency motor cortex inhibition. *Exp. Brain Res.* 198 489–500. 10.1007/s00221-009-1945-8 19618169

[B59] Stinear C. M. Petoe M. A. Byblow W. D. (2015). Primary motor cortex excitability during recovery after stroke: implications for neuromodulation. *Brain Stimul.* 8 1183–1190. 10.1016/j.brs.2015.06.015 26195321

[B60] Thickbroom G. W. Byrnes M. L. Stell R. Mastaglia F. L. (2003). Reversible reorganisation of the motor cortical representation of the hand in cervical dystonia. *Mov. Disord.* 18 395–402. 10.1002/mds.10383 12671945

[B61] Tomasova Z. Hlustik P. Kral M. Otruba P. Herzig R. Krobot A. (2013). Cortical activation changes in patients suffering from post-stroke arm spasticity and treated with botulinum toxin a. *J. Neuroimaging* 23 337–344. 10.1111/j.1552-6569.2011.00682.x 22212022

[B62] Ward A. B. (2012). A literature review of the pathophysiology and onset of post-stroke spasticity. *Eur. J. Neurol.* 19 21–27. 10.1111/j.1468-1331.2011.03448.x 21707868

[B63] Ward N. S. Brown M. M. Thompson A. J. Frackowiak R. S. (2003). Neural correlates of motor recovery after stroke: a longitudinal fMRI study. *Brain* 126 (Pt 11), 2476–2496. 10.1093/brain/awg245 12937084 PMC3717457

[B64] Wittenberg G. F. Bastings E. P. Fowlkes A. M. Morgan T. M. Good D. C. Pons T. P. (2007). Dynamic course of intracortical TMS paired-pulse responses during recovery of motor function after stroke. *Neurorehabil. Neural Repair* 21 568–573. 10.1177/1545968307302438 17522261

[B65] Ziemann U. Chen R. Cohen L. G. Hallett M. (1998). Dextromethorphan decreases the excitability of the human motor cortex. *Neurology* 51 1320–1324. 10.1212/wnl.51.5.1320 9818853

[B66] Ziemann U. Ishii K. Borgheresi A. Yaseen Z. Battaglia F. Hallett M. (1999). Dissociation of the pathways mediating ipsilateral and contralateral motor-evoked potentials in human hand and arm muscles. *J. Physiol.* 518 (Pt 3), 895–906. 10.1111/j.1469-7793.1999.0895p.x 10420023 PMC2269467

[B67] Ziemann U. Lonnecker S. Paulus W. (1995). Inhibition of human motor cortex by ethanol. A transcranial magnetic stimulation study. *Brain* 118 (Pt 6), 1437–1446. 10.1093/brain/118.6.1437 8595475

[B68] Ziemann U. Lonnecker S. Steinhoff B. J. Paulus W. (1996a). The effect of lorazepam on the motor cortical excitability in man. *Exp. Brain Res.* 109 127–135. 8740215 10.1007/BF00228633

[B69] Ziemann U. Rothwell J. C. Ridding M. C. (1996b). Interaction between intracortical inhibition and facilitation in human motor cortex. *J. Physiol.* 496 (Pt 3), 873–881. 10.1113/jphysiol.1996.sp021734 8930851 PMC1160871

[B70] Zorowitz R. D. Gillard P. J. Brainin M. (2013). Poststroke spasticity: sequelae and burden on stroke survivors and caregivers. *Neurology* 80 (3 Suppl. 2), S45–S52. 10.1212/WNL.0b013e3182764c86 23319485

